# Why F-ATP Synthase Remains a Strong Candidate as the Mitochondrial Permeability Transition Pore

**DOI:** 10.3389/fphys.2018.01543

**Published:** 2018-11-01

**Authors:** Paolo Bernardi

**Affiliations:** Department of Biomedical Sciences, University of Padua, Padua, Italy

**Keywords:** mitochondria, ATP synthase, permeability transition pore, channels, calcium

## The permeabiliy transition and F-ATP synthase

Mitochondria can undergo a Ca^2+^-dependent increase of inner membrane permeability called the permeability transition (PT; Hunter et al., 1976). The PT requires Ca^2+^ accumulation in the matrix and is due to opening of a regulated channel, the PT pore (PTP) which has also been studied by electrophysiology and named mitochondrial megachannel (MMC; Kinnally et al., 1989; Petronilli et al., 1989). The PTP/MMC displays a range of conductance states, which also depend on the species. In mammals maximal conductance can be as high as 1.2 nS, which corresponds to a pore with a diameter of about 2–3 nm. The channel is characterized by a variety of lower conductance states, which may allow selective permeation of small solutes (Szabo and Zoratti, 2014). In mammals the PTP is modulated by binding of cyclophilin D, which favors PTP opening. The molecular nature of the PTP/MMC is the matter of active investigation and controversy, and has been specifically addressed in a recent review (Bernardi and Lippe, 2018). The most recent hypothesis posits that it originates from a conformational change occurring on the F-ATP synthase after Ca^2+^ binding, possibly by replacing Mg^2+^ at the catalytic site (Giorgio et al., 2017). This proposal has been supported by genetic manipulation of F-ATP synthase (Bonora et al., 2013; Giorgio et al., 2013), by electrophysiological measurements (Giorgio et al., 2013; Alavian et al., 2014; Carraro et al., 2014; von Stockum et al., 2015), and by mutagenesis of specific residues of F-ATP synthase (Giorgio et al., 2017; Antoniel et al., 2018; Guo et al., 2018; Carraro et al., in press). On the other hand, the Walker laboratory has challenged this hypothesis on the basis of studies where subunit c (He et al., 2017b) or peripheral subunits b and OSCP (He et al., 2017a) had been genetically ablated. These studies are important because they provide the first example of eukaryotic cells where F-ATP synthase subunits have been genetically turned off, thus allowing the first appraisal of the consequences of F-ATP synthase loss of function (He et al., 2017a,b). The Authors also used these cells to address the question of whether the PTP is conserved. They concluded that the PTP was still present in the deletion mutants, and claimed that the idea that F-ATP synthase is an essential component of the PTP can be ruled out (He et al., 2017a,b). However, analysis of the data suggests that the PTP has been affected by elimination of subunits c, b and OSCP, and that the above conclusion needs to be reassessed.

## Size matters

The PTP size is large enough for the diffusion of sucrose, which is the typical solute used to detect occurrence of a PT. Long-lasting PTP opening *in vitro* is followed by solute diffusion with matrix swelling (Massari and Azzone, 1972). Swelling obviously also occurs in media based on KCl or other salts, conditions that allow detection of pore(s) of smaller size and/or of lower conductance (Ichas et al., 1997). He et al. observed that Ca^2+^-dependent mitochondrial swelling took place in permeabilized wild-type cells as well as in cells obtained after genetic ablation of subunits b (Δb) and OSCP (ΔOSCP) (He et al., 2017a). However, analysis of the data reveals that, compared to wild-type cells, the rate of swelling was reduced to about 20% in the Δb and to 40% in the ΔOSCP cells. This finding may not be immediately appreciated because the ordinate scale of the latter two sets of experiments was expanded (Figure 5 of He et al., 2017a). The absence of replicates and of calibration with pore-forming agents like alamethicin prevents firm conclusions to be drawn from these experiments. However, decreased swelling rates in KCl would support the conclusion that in the deletion mutants the PTP size has become smaller, and close to the exclusion size of hydrated K^+^ and Cl^−^, a difference that would have been even more dramatic in sucrose-based media. In a second set of experiments occurrence of PTP opening was determined based on the Ca^2+^ load required to trigger Ca^2+^ release after the accumulation of a train of Ca^2+^ pulses, the so-called Ca^2+^ retention capacity (CRC). In these protocols Ca^2+^ release (which marks onset of the PT) is due to depolarization and does not provide information on the mechanism(s) mediating depolarization itself, which could result even from selective permeabilization to H^+^ as seen in Ca^2+^ release experiments induced by the addition of protonophores (Bernardi et al., 1984). These experiments are therefore also compatible with opening of a PTP of smaller size.

## Knock out of F-ATP synthase subunits severely impairs respiration

As a consequence of the ablation of subunits c, b, and OSCP F-ATP synthase was not properly assembled and rather originated a “vestigial” enzyme that did not display dimer formation after extraction with digitonin. Remarkably, major alterations were also observed in the respiratory chain with almost no mature complex I assembly, severe depletion of complex III and reduction of complex IV. Consistently, respiratory activity was dramatically decreased to between 10 and 20% of the rate observed in wild-type cells, and could only be marginally stimulated by uncoupler (He et al., 2017a,b). Mitochondrial Ca^2+^ uptake, which is essential for PTP opening, is driven by the Ca^2+^ electrochemical gradient ($\Delta \tilde{\mu }$Ca = zFΔψ + RT ln [Ca^2+^]_i_/[Ca^2+^]_o_). In respiring mitochondria the driving force for accumulation is the inside-negative membrane potential generated by respiration, and Ca^2+^ uptake takes place on the uniporter with net charge translocation of 2 (Scarpa and Azzone, 1970; Wingrove et al., 1984; Kirichok et al., 2004). Ca^2+^ uptake is charge-compensated by increased H^+^ pumping by the respiratory chain, while the buildup of a large ΔpH and of high matrix Ca^2+^ concentration is prevented by Pi uptake. The essential point for the present discussion is that the maximal rate of Ca^2+^ uptake is limited by the maximal rate of H^+^ pumping by the respiratory chain (Bragadin et al., 1979), the latter becoming rate-limiting when extramitochondrial Ca^2+^ rises above about 2 μM (Nicholls, 1978). Given that the size of the Ca^2+^ pulses was 10 μM (He et al., 2017a,b) the rate of Ca^2+^ uptake in the Δc, Δb, and ΔOSCP mitochondria should have been measurably lower than (rather than identical to) the rate observed in wild-type mitochondria. It is legitimate to ask whether the marked respiratory inhibition described for the deletion mutants is constant over time, or rather compensatory mechanisms exist that eventually restore at least partial expression of F-ATP synthase and of the respiratory chain.

A good example is provided by the Δb mutants. The Authors report the surprising finding that low levels of tryptic peptides corresponding to sequences of subunit b could still be found in the null cells. The peptides were derived from a gel region at 17.5 kDa, which corresponds to truncated subunit b. PCR amplification of RNA transcripts covering a putative coding region for these peptides revealed the existence of an alternative splice site in intron A allowing generation of a truncated subunit b (residues 67–214) lacking the membrane region (He et al., 2017a). In summary, the swelling experiments of the Walker laboratory suggest that deletion of the c and peripheral stalk subunits may have affected the PTP, which appears to have become smaller; and contain an internal inconsistency between inhibition of respiration and rates of Ca^2+^ uptake, which in turn raises questions about the CRC measurements. Thus, these results cannot be used to conclude that the F-ATP synthase does not take part in formation of the PTP.

## What we have learned from site-directed mutagenesis

Several sites have been defined by chemical modification with relatively selective sulfhydryl, histidine and arginine reagents that confer PTP regulation by the membrane potential, matrix pH, divalent cations, quinones, and oxidative stress. Identification of these sites is a formidable challenge but also a unique opportunity to (dis)prove our hypothesis on the identity of the PTP. Indeed, site-directed mutagenesis of specific residues should modify the properties of the PTP in a predictable manner, a task that is made easier by the availability of structures of F-ATP synthase of increasing resolution (Abrahams et al., 1994; Stock et al., 1999; Rubinstein et al., 2003; Strauss et al., 2008; Baker et al., 2012; Davies et al., 2012; Daum et al., 2013; Allegretti et al., 2015; Jiko et al., 2015; Zhou et al., 2015; Hahn et al., 2016; Vinothkumar et al., 2016; Guo et al., 2017).

Residue T163 in the β subunit of mammalian F-ATP synthase is essential for the binding of Mg/ADP to the catalytic site (Rees et al., 2012). Mg^2+^ can be replaced by other divalent metals (Selwyn, 1968; Pedersen et al., 1987), and binding of Ca^2+^ allows ATP hydrolysis without measurable H^+^ pumping (Papageorgiou et al., 1998). In *Rhodospirillum rubrum* the relative affinity for Ca^2+^ and Mg^2+^ could be modulated with a T159S mutation at the β subunit (the position equivalent to T163 in mammals), which led to decreased Ca^2+^-ATPase and increased Mg^2+^-ATPase activities (Nathanson and Gromet-Elhanan, 2000; Du et al., 2001). We have found that a partial T163S substitution in HeLa cells increases Mg^2+^-ATP and prevents Ca^2+^-ATP hydrolysis with a matching decreased sensitivity of the PTP to Ca^2+^, resistance to cell death and decreased apoptosis (Giorgio et al., 2017). One of the most remarkable features of the PTP is its absolute dependence on matrix Ca^2+^, and these findings are consistent with the idea that the PTP is triggered by Ca^2+^ binding at the F-ATP synthase catalytic site.

Previous studies had identified a role of Arg residues in the regulation of the PTP through the use of selective reagents such as phenylglyoxal (PGO; Johans et al., 2005). The effects of PGO are species-specific, and we recently identified R107 of F-ATP synthase subunit g as the unique PTP-modulating target of PGO in yeast. Remarkably, expression of human subunit g in yeast transferred the “human” PTP phenotype, suggesting that species-specificity depends on differences in the primary structure of F-ATP synthase (Guo et al., 2018). The importance of the e and g subunits in formation of the yeast channel is also supported by our recent finding that their deletion abrogated the high-conductance channels in mutants where dimerization was enforced by copper-dependent formation of dimers through oxidation of C23 of subunit a (Carraro et al., in press).

Perhaps the most intriguing feature of the PTP is its inhibition by matrix H^+^, which is marked at pH 6.7 and leads to complete channel block at pH 6.5 (Nicolli et al., 1993; Antoniel et al., 2018). Based on protection with diethylpyrocarbonate and partial reversal with hydroxylamine, we had concluded that PTP block is mediated by reversible protonation of matrix-accessible His residues (Nicolli et al., 1993). We have recently identified H112 of the OSCP subunit as the unique His responsible for the PTP block by H^+^. Indeed, H112Q and H112Y mutations completely prevented the inhibitory effects of acidic pH both in PTP-dependent swelling measurements in mitochondria and in single-channel patch-clamp recordings in mitoplasts (Antoniel et al., 2018). Remarkably, the mutations had no consequences on oligomycin-sensitive and uncoupler-stimulated respiration, indicating that the F-ATP synthase was normally assembled.

At variance from knock out experiments, our mutagenesis approach turned out not to be disruptive for F-ATP synthase assembly and catalysis, nor to have detectable effects on respiration and cell viability. We are confident that future work will allow a better understanding of how the energy-conserving enzyme can turn into an energy-dissipating device, a hypothesis that stands and that will be further tested by mutagenesis of Cys residues and by analysis of the channel activity of highly purified preparations from bovine heart.

## Author contributions

The author confirms being the sole contributor of this work and has approved it for publication.

### Conflict of interest statement

The author declares that the research was conducted in the absence of any commercial or financial relationships that could be construed as a potential conflict of interest.
